# The role of oncogenes and tumor suppressor genes in determining survival rates of lung cancer patients in the population of North Sumatra, Indonesia

**DOI:** 10.12688/f1000research.113303.1

**Published:** 2022-07-28

**Authors:** Noni Novisari Soeroso, Fannie Rizki Ananda, Johan Samuel Sitanggang, Noverita Sprinse Vinolina

**Affiliations:** 1 Thoracic Oncology Division, Department of Pulmonology and Respiratory Medicine, Universitas Sumatera Utara, Medan, Sumatera Utara, 20155, Indonesia; 2 Department of Pulmonology and Respiratory Medicine, Universitas Sumatera Utara, Medan, Sumatera Utara, 20155, Indonesia; 3 Faculty of Medicine, Universitas Sumatera Utara, Medan, Sumatera Utara, 20155, Indonesia; 4 Department of Statistics, Universitas Sumatera Utara, Medan, Sumatera Utara, 20155, Indonesia

**Keywords:** lung cancer, EGFR, KRAS, TP53, survival rate.

## Abstract

**Background:** Gaining a better understanding of molecular alterations in the pathogenesis of lung cancer reveals a significant change in approach to the management and prognosis of lung cancer. Several oncogenes and tumor suppressor genes have been identified and have different roles related to survival rates in lung cancer patients. This study aims to determine the role of KRAS, EGFR, and TP53 mutations in the survival rate of lung cancer patients in the population of North Sumatra.

**Methods:** This is a retrospective cohort study involving 108 subjects diagnosed with lung cancer from histopathology specimens. DNA extractions were performed using FFPE followed by PCR examinations for assessing the expressions of EGFR, RAS, and TP53 protein. Sequencing analysis was carried out to determine the mutations of EGFR exon 19 and 21, RAS protein exon 2, and TP53 exon 5-6 and 8-9. Data input and analysis were conducted using statistical analysis software for Windows. The survival rate analysis was presented with Kaplan Meier.

Results:

52 subjects completed all procedures in this study. Most of the subjects are male (75%), above 60 years old (53.8%), heavy smokers (75%), and suffer from adenocarcinoma type of lung cancer (69.2%). No subjects showed KRAS exon 2 mutations. Overall survival rates increased in patients with EGFR mutations (15 months compared to 8 months; *p*=0.001) and decreased in patients with TP53 mutations (7 months compared to 9 months; *p*=0.148). Also, there was increasing Progression-Free Survival in patients with EGFR mutations (6 months compared to 3 months) ( *p*=0.19) and decreasing PFS in patients with TP53 mutations (3 months compared to 6 months) ( *p*=0.07).

**Conclusions:** There were no KRAS mutations in this study. EGFR mutations showed a higher survival rate, while TP53 mutations showed a lower survival rate in overall survival and progression-free survival.

## Background

Recent molecular studies have focused on the genetic alterations and epigenetic impacts in the pathogenesis of lung cancer. ^1^ Several tumor suppressor genes and oncogenes have been identified and altered the micro and macro environment related to lung carcinogenesis. ^2^ This was a multistep process where the oncogenes mutation initiates and triggers conversions of the normal epithelium to cancer cells due to environmental factors, particularly cigarette smoke. ^1^ P53 and KRAS genes play crucial roles in maintaining cells integrity that affects the early stage of lung carcinogenesis, particularly in lung cancer-related tobacco exposure. ^3^ Recent studies showed the mutations of KRAS and TP53 mutations have been related to shorter overall survival and progression-free survival rates in lung cancer patients, whether in early or advanced stages. ^4^^,^^5^ In contrast, EGFR mutations, as one of the most common oncogenic mutations together with targeted therapy management, gives longer survival times than for subjects with wild-type mutations who received systemic chemotherapy. ^6^^–^^8^

However, there has been a surge of EGFR resistance around the world. The concurring mutations with other oncogenes and tumor suppressor genes have been identified as the cause of EGFR resistance. ^3^^–^^5^^,^^9^^,^^10^ Unfortunately, there is still limited data regarding oncogenes and tumor suppressor gene mutations in Indonesia. This study aimed to assess the role of oncogenes and tumor suppressor genes in determining the survival rate of lung cancer patients in the population of North Sumatra.

## Methods

### Research design and participants

This was a retrospective cohort study conducted in the Department of Pulmonology and Respiratory Medicine, Faculty of Medicine, Universitas Sumatra Utara during the period of 2017–2020. All subjects were enrolled in the study via several cancer-referral hospitals in North Sumatra in collaboration with the Faculty of Medicine, Universitas Sumatra Utara. The criteria for inclusion in this study were subjects aged 18 or over and diagnosed with lung cancer confirmed by histopathology preparations from bronchoscopy, open biopsy, thoracotomy, and trans-thoracal lung biopsy. The number of cells in one section of the paraffin block must be a minimum of 50 cells. All medical records were then observed to assess the demographic and clinical characteristics and followed the progressivity of disease based on RECIST criteria. ^11^ Subjects’ deaths were recorded in their medical records in the case of subjects who passed away in hospital, while others were assessed based on phone calls from the subjects’ relatives addressed in medical records. The exclusion criteria of this study were an inadequate amount of cancer cells in a paraffin block, incomplete medical records, and difficulties in assessing the subjects’ relatives.

All the procedures of this study were approved by the Ethics Committee of the Faculty of Medicine, Universitas Sumatra Utara with reference number 124/KEP/USU/2020. Written informed consent was obtained from all the subjects or their relatives. All the pathology specimens received permission for genetic analysis from the Pathology Department at each collection site.

### Molecular testing

DNA was extracted from Formalin-Fixed Paraffin-Embedded using Quick-DNA FFPE mini prep (Zymo Research, USA) and sliced using microtome in 1–2 slices and 4 μm thickness. After being deparaffinized, digested, and purified, the DNA was eluted by a DNA elution buffer. The PCR was performed using MyTaq HS Red Mix (Bioline, UK) with the following process: denaturation, annealing, and elongation. DNA was then amplified using certain primers ( Table 1) and run in 2% agarose electrophoresis in Tris Buffer EDTA pH 8.0. Sequencing analysis was carried out via two different methods. EGFR and KRAS were converted to reverse complement and analysed using ClustalW in BioEdit Sequence Alignment Editor 7.2.5. The reverse sequence was also compared to human reference genome NG_007524.2 (LRG_433) using BLASTn NCBI. The BLASTn results were also aligned with corresponding Coding Sequences (CDS). Lastly, the sequence was analysed using FinchTv 1.4 Software using reverse complement views. Meanwhile, TP53 genes were analysed using Unipro Ugene v. 38.1 software (RRID: SCR_005579) and compared with standard reference NG-017013.2. Samples in which there was a change in protein base analysed using *Nucleotide Basic Local Alignment Search Tool* (BLASTn) were then compared with several TP53-specific databases including SNP (dbSNP), Cosmic (assessing somatic mutation), ClinVar (Clinical variations, assessing the correlation of mutations with clinical profile).

**Table 1.** Primer used in examining EGFR, KRAS, and EGFR.

Primer Name	PRIMER	Primer Sequence (5′–3′)	Primer Concentration (μM)	Amplicon Size (bp)
KRAS	Forward	AAGGCCTGCTGAAAATGACTG	20	173
	Reverse	CAAAGAATGGTCCTGCACCAG		
TP53 EXON 5-6	Forward Reverse	5′-GTTGCAGGAGGTGCTTACG-3′ 5′-CATGGGGTTATAGGGAGGTC-3′	20	606
TP53 EXON 8-9	Forward Reverse	5′-GACAAGGGTGGTTGGGAGTAG-3′ 5′-CCAGGAGCCATTGTCTTTGAG-3′	20	546
EGFR EXON 19	Forward	TCACTGGGCAGCATGTGGCA	20	241
	Reverse	CAGCTGCCAGACATGAGAAA		
EGFR EXON 21	Forward	AGAGCCTGGCATGAACATGA	20	370
	Reverse	CGAGCTCACCCAGAATGTCT		
EGFR 21 ARMS PCR	Forward (F)	AGGGTCTTCTCTGTTTCAGGGCAT	10	
	Reverse T (T)	TTCCGCACCCAGCAGTTTGGCTA		FT: 137
	Reverse G (G)	CGCACCCAGCAGTTTGGTTC		FG: 134

## Results

**Figure 1.**
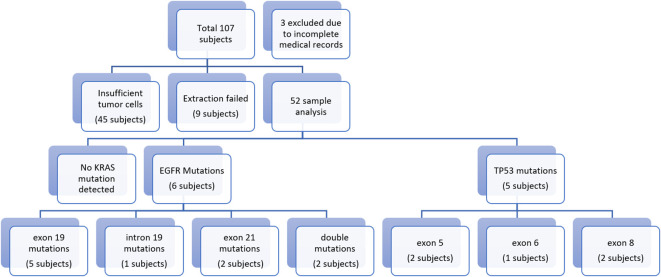
Flowchart of research results.

As shown in Figure 1, a total of 52 subjects completed the procedures of this study with most of the subjects being male (75%), aged over 60 years (53.8%), heavy smokers (75%), with adenocarcinoma type lung cancer (69.2%) stage IVA (71.25%). Further characteristics were as described in Table 2. From sequencing analysis results no KRAS exon 2 mutations were detected, while EGFR mutations were detected in 6 subjects, consisting of 5 subjects who had exon 19 mutations, 1 subject with intron 19 mutations, and 2 subjects with exon 21 mutations. TP53 mutations were seen in 5 subjects, of which 2 subjects had missense mutations of exon 5, 1 subject showed silent mutations, 1 subject with missense exon 8mutations, and 1 subject had nonsense exon 8 mutations.

**Table 2.** General characteristics of population.

No.	Characteristics	N	%
1	Sex		
	Female	12	25
	Male	40	75
2	Smoking Habits		
	Non-smoker	7	13.5
	Light smoker	6	11.5
	Heavy Smoker	39	75
3	Age		
	<40 years old	1	1.9
	40-60 years old	23	44.2
	>60 years old	28	53.8
4	Histopathology type		
	Adenocarcinoma	36	69.2
	Squamous Cell	12	23.1
	Small Cell Carcinoma	4	7.7
5	Staging		
	IIIB	12	23.1
	IIIC	3	5.8
	IVA	37	71.2

**Figure 2.**
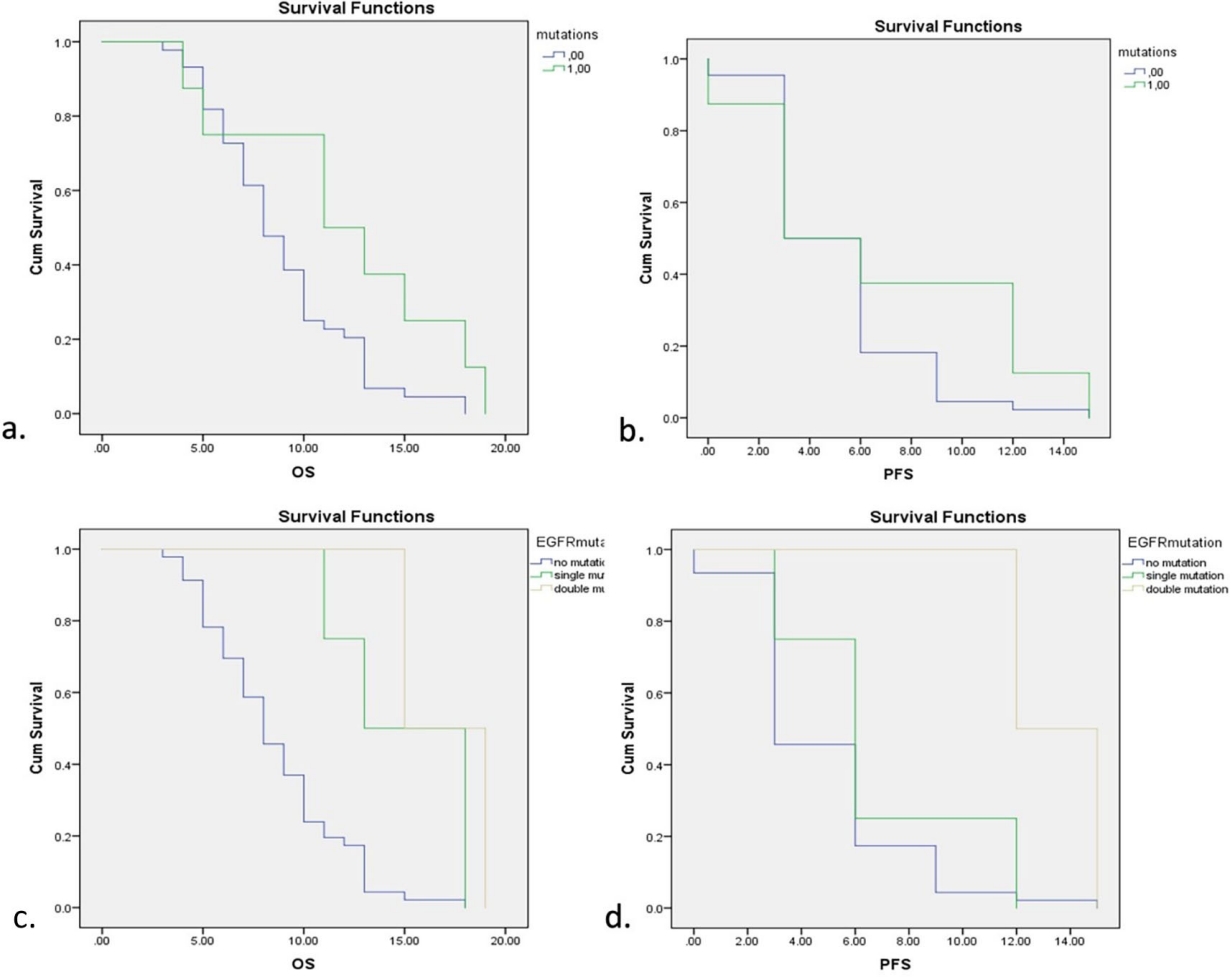
Survival analysis for EGFR mutations. a. Overall Survival (OS) with and without EGFR mutations. b. Progression-free Survival (PFS) with and without EGFR mutations. c. OS with different types of EGFR mutations. d. PFS with different types of EGFR mutations.

Survival analysis for EGFR mutations is depicted in Figure 2. EGFR mutations showed a significant increase in both overall survival (OS) and progression-free survival (PFS) with *p*=0.001 for OS and *p*=0.018 for PFS (Log-Rank Test). Five of six subjects showed mutations in exon 19 and 2 subjects showed double mutations in exon 19 and exon 21. Further analysis also showed significant improvements in OS and PFS of both single and double mutations of EGFR while double mutations showed higher OS and PFS compared with single mutations. Detailed comparisons of EGFR mutations are described in Table 3.

**Table 3.** Comparisons of overall survival and progression-free survival among subjects with oncogenes and tumor suppressor genes.

Mutations	Overall survival	*p*-value	Progression-free survival	*p*-value
EGFR mutations	15	0.001 *	6	0.018 *
Exon 19	15		6	
Intron 19	18		12	
Exon 21	15		12	
Double mutations	15		12	
TP53 mutations	7	0.148	3	0.070
Exon 5	9		3	
Exon 6	5		3	
Exon 8	7		6	
Missense	7		3	
Silent	5		3	
Nonsense	9		6	
No mutations	8	0.037 *	3	0.196

Log Rank Test.

* *p*-value < 0.05 considered significant.

**Figure 3.**
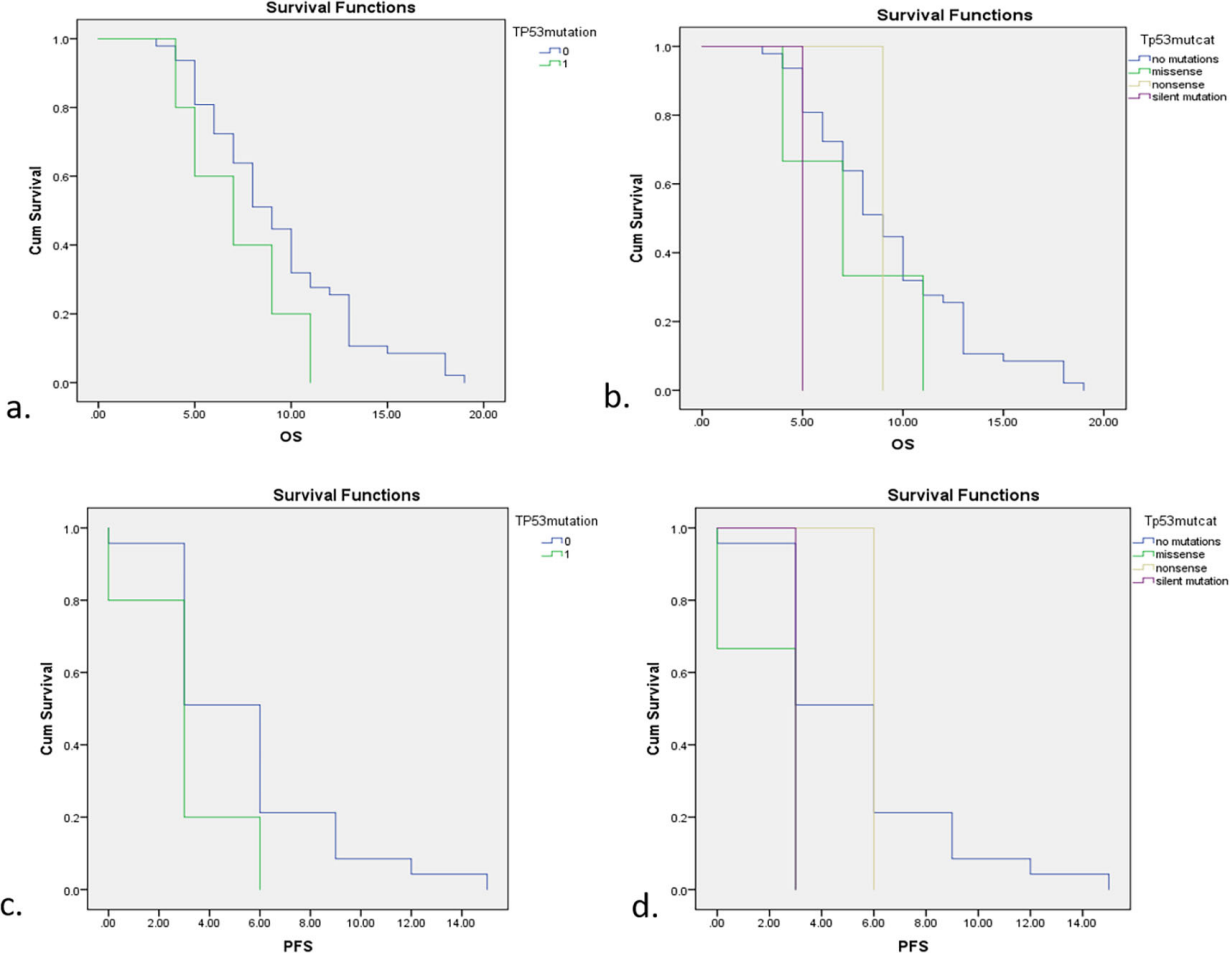
Survival analysis according to TP53 mutations status. a. OS between subjects with and without TP53 mutations. b. OS with different types of TP53 mutation. c. PFS between subjects with and without TP53 mutations. d. PFS with different types of TP53 mutation.

Survival analysis was presented in OS and PFS curve in Figure 3. Generally, subjects with TP53 mutations had a lower survival rate including OS and PFS compared with non-mutations. Subjects with TP53 mutations had 7 months of OS, 2 months less than subjects without TP53 mutations ( *p*=0.148). This study also compared median survival rates for different types of TP53 mutation including non-mutations, missense mutations, nonsense mutations, and silent mutations as follows; 9 months, 7 months, 9 months, and 5 months. Nevertheless, the association between median survival rates and different types of TP53 mutation was not significant in statistical analysis with a *p*-value of 0.245. Patients with TP53 mutations also had a three months’ shorter PFS compared to those with non-mutations (3 months vs 6 months; *p*=0.07). After further analysis according to different types of mutation, the PFS of subjects with missense mutations and silent mutations was 3 months, while for subjects with non-mutations and nonsense mutations it was 6 months. These different mutations were also insignificant in relation to median PFS with a *p*-value of 0.107.

The survival rates assessed in this study were overall survival (OS) and progression-free survival (PFS) and Table 3 shows the comparisons of OS and PFS in subjects with EGFR, TP53, and no mutations. EGFR mutations showed a significant increase in both OS and PFS compared with no mutations, while TP53 was a poor predictor of lung cancer patients with shorter survival rates.

## Discussion

KRAS mutation is one of the common oncogenes mutations that are related to poor prognosis of lung cancer and is strongly associated with smoking. ^12^^–^^19^ This study follows on from a study by Soeroso *et al.* which analyzed KRAS mutations in the population of North Sumatra. ^20^ However, there were no KRAS exon 2 mutations among the subjects of this study. The scanty population of KRAS mutations in Asia is not completely understood, although recent data has shown that Asia has a lower prevalence of KRAS mutations compared with worldwide data. ^21^^,^^22^ In Asian populations, KRAS mutations were present in 4.3%–10.5% of the population, while data shows that KRAS mutations were present in 30–40% of populations worldwide. ^23^ In addition, Syahruddin *et al.*, as the first study to identify KRAS mutations in lung cancer in Indonesia, showed a lower incidence of KRAS mutations compared with populations worldwide (7% vs 31%). ^24^^,^^25^ The occurrence of KRAS mutations also often coincides with mutations in other oncogenes or tumor suppressor genes. Co-mutations between KRAS and TP53 were the most prevalent and were related to the poorest outcomes in all pharmacological approaches including targeted therapy, chemotherapy, and radiotherapy. ^25^ However, studies by Fang *et al.* and Dong *et al.* showed a better outcome in administering immune checkpoint inhibitors in a patient with KRAS and TP53 mutations compared with KRAS mutations alone or with no mutations at all. ^26^^,^^27^ On the other hand, KRAS mutations were among the most common factors related to EGFR-TKI resistance. Several studies reported the concurring EGFR-KRAS mutations in 15–20% populations ^28^^–^^31^ while KRAS mutations were presumed to be independent factors in resistance to EGFR-TKI treatment. The absence of KRAS mutations in this study directs the pulmonologists to analyze other factors which might contribute to resistance to EGFR-TKI treatment in the population of North Sumatra and other populations with a low prevalence of KRAS mutations, despite the limitations of facilities in detecting KRAS mutations in certain populations.

KRAS mutations can be detected in cytology and histopathology examinations following the RT-PCR or DNA Sequencing method. ^32^ The type of KRAS mutation will be identified using the DNA-Sequencing method where a change in GGT to TTG is addressed as G12C, ^33^ the most common mutation of KRAS related to longer survival rate compared with other mutations. ^25^ The second most common KRAS mutation type is in codon 12 changes in GGT to GTT, referred to as G12V mutations. ^33^ In this study, we examined the histopathology preparations from the paraffin block for all subjects diagnosed with lung cancer, and from the PCR test it was found that almost all subjects showed expressions of RAS protein in exon 2 codon 12-13. Nevertheless, there was no change in protein base with DNA sequences showing GGT-GGC known as normal RAS protein expressions. This finding showed that the Sanger sequencing method carried out in this study is still reliable in identifying the KRAS mutations, although the variations in North Sumatra populations showed no mutations.

Role of the EGFR mutations in lung cancer has been described in previous studies. ^8^^,^^34^^,^^35^ EGFR mutations treated with EGFR-TKI-targeted therapy showed a significantly improved survival rate with lung adenocarcinoma. ^7^^,^^35^^,^^36^ However, the multiple mutations are not completely understood. The various studies tried to identify the impact of double mutations or triple mutations of EGFR in clinicopathology characteristics in lung cancer patients. ^37^^,^^38^ All previous studies showed poorer outcomes with multiple mutations compared with single EGFR mutations. ^37^^,^^38^ Nevertheless, in this study, double mutations of exon 19 and exon 21 showed a better outcome in both OS and PFS compared with single mutations of exon 19. Unfortunately, this study was a small size study, so it is still difficult to draw general conclusions concerning the wider population. In the previous studies, the double mutations consisted of exon 20 T790M, which has been identified as primary factors in 1 ^st^ and 2 ^nd^ EGFR TKI-resistance. ^38^ Barnet *et al.* also showed shorter PFS in a patient with co-mutations of exon 19 with noncommon EGFR mutations including exon 20, T90M, and PIK3CA. ^39^ This differs from the current study, which looked at exon 19 and exon 21 mutations. A larger scale of genetic studies is needed to evaluate the impact of double mutations in the treatment of lung adenocarcinoma, both in receiving targeted therapy and systemic chemotherapy.

In addition to the oncogenes mutations mentioned above, mutations of TP53 as one of the most common tumor suppressor gene mutations associated with resistance to cancer therapy. ^40^^,^^41^ Having a role as the guardian of the genome, the TP53 gene maintains the integrity of the genome by modifying, stabilizing, and preventing cell proliferation in response to cellular stress. ^42^^–^^45^ This study found five subjects with TP53 mutations and showed a shorter survival rate in both OS and PFS. When different kinds of mutation were analyzed in depth, missense and silent mutations showed shorter OS and PFS compared with nonsense mutations. However, the small number of subjects analyzed in this study might bias the effect due to individual variations. On a larger scale of different types of TP53, a missense mutation is the most common TP53 mutation, ^46^ particularly in tobacco-related lung cancer. ^46^^,^^47^ According to the latest evidence, missense mutation has a “gain-of-functions” effect, leading to an increase in the expression of cancer cells. ^48^^,^^49^ In contrast, Halvorsen found that missense mutations showed higher OS compared with other types of TP53 mutations although, in general, TP53 mutations showed a higher Hazard Ratio (HR) compared with no mutations. ^46^

As oncogene activations occur, TP53 activates and arrests the cell cycle in both G1 and G2, allowing sufficient time for DNA repair. ^50^^,^^51^ Along with RAS mutations, p53 is a multifunctional protein which alters cell regulations, cancer cell transformations, and vascular invasion in all solid cancers including lung cancers. Therefore, oncogenic activations and over-expressions of p53 genes are independent predictors of poor prognosis in NSCLC. In this study, there were no co-mutations of TP53 with any of EGFR or RAS mutations meaning it is difficult to evaluate the interactions of concurring mutations, whether they have mutual or opposite effects.

Other data highlighted in this study is the lower number of mutations of oncogene and tumor suppressor gene in North Sumatra. A large cohort study in Norway showed 38% of subjects had positive mutations of KRAS in non-small cell lung cancer patients, while in Chen’s study involving a lower number of subjects KRAS mutations accounted for 11% of all mutations with a higher prevalence among smokers (20–30%) compared with those who never smoked (7–13%). ^52^ This is in contrast this to study which found no KRAS mutations in subjects diagnosed with lung cancer. EGFR was the most common oncogene mutation, particularly in Asia. The median global prevalence of EGFR in the advanced stage of NSCLC was 33.07% ^53^ while 47% of Asians showed EGFR mutations. ^52^ The latest data on EGFR mutations in Indonesia also revealed EGFR mutations in 44.4% of 1874 cytological specimens diagnosed with EGFR. In this study, just 11.5% of subjects were positive with EGFR mutations with allele-specific for exon 19 and 21 and DNA sequencing. Furthermore, TP53, as the common tumor suppressor gene accounted for more than 50% in NSCLC ^40^^,^^52^^,^^54^ was only detected in 9.6% of the subjects in this study. A definite mechanism explaining the lower prevalence of both oncogenes and tumor suppressor genes may not be understood yet. Lung carcinogenesis is a complex process characterized by the after-effects of genetic variations as the individual process works synergistically with environmental exposure. ^55^^,^^56^ Long durations of exposure to carcinogens might alter DNA methylation, resulting in the conversion of normal cells to cancer cells. Therefore, a better understanding of genetic variations and individual vulnerability of lung carcinogenesis may provide better anti-cancer research.

Although this study has several limitations including the small samples size and the limited survival aspect, this is the first study to identify TP53 mutations in lung cancer patients in Indonesia and the first study to identify the DNA sequencing of common oncogenes and tumor suppressor genes in the population of Sumatra. Further multi-center study involving larger sample size is needed to identify the prevalence of oncogenes and tumor suppressor genes to actualized personal therapeutic approach for cancer patients, particularly in a developing country such as Indonesia.

## Data availability

### Underlying data

Figshare: Underlying data for “The role of oncogenes and tumor suppressor genes in determining survival rates of lung cancer patients in the population of North Sumatra, Indonesia”, https://doi.org/10.6084/m9.figshare.19522468. ^57^

In addition, this study adds Research Resource Identifiers (RRIDs) from these research resources including the following software tools and data:

1. Human lung carcinoma (RRID:CVCL_5791)
https://web.expasy.org/cellosaurus/CVCL_5791
2. FFPE/Formalin-Fixed Paraffin-Embedded (RRID:SCR_001307)
http://www.bioconductor.org/packages/release/bioc/html/ffpe.html
3. FinchTV (RRID:SCR_005584)
http://www.geospiza.com/Products/finchtv.shtml
4. ClustalW (RRID:SCR_017277)
http://clustalw.ddbj.nig.ac.jp/index.php
5. BLASTn (RRID:SCR_001598)
http://blast.ncbi.nlm.nih.gov/Blast.cgi?PROGRAM=blastn&BLAST_PROGRAMS=megaBlast&PAGE_TYPE=BlastSearch
6. Unipro UGENE (RRID:SCR_005579)
http://ugene.unipro.ru/
