# Supplementary material for: The role of oncogenes and tumor suppressor genes in determining survival rates of lung cancer patients in the population of North Sumatra, Indonesia
Source: F1000Res. 2023 Jun 15;11:853. Originally published 2022 Jul 28. [Version 2] doi: 10.12688/f1000research.113303.2 (PMC10329197; doi:10.12688/f1000research.113303.2)
Supplement: Peer review history

# Peer review: Reviewer response for version 2

**DOI:** 10.5256/f1000research.151020.r178874

**Published:** 2023-07-07

**Authors:** Darren Wan-Teck Lim

**Affiliations:** 1 Division of Medical Oncology, National Cancer Centre Singapore, Singapore, Singapore

The authors have addressed my concerns appropriately.

Is the work clearly and accurately presented and does it cite the current literature?

Yes

If applicable, is the statistical analysis and its interpretation appropriate?

Yes

Are all the source data underlying the results available to ensure full reproducibility?

Yes

Is the study design appropriate and is the work technically sound?

Partly

Are the conclusions drawn adequately supported by the results?

Partly

Are sufficient details of methods and analysis provided to allow replication by others?

Yes

Reviewer Expertise:

Lung cancer

I confirm that I have read this submission and believe that I have an appropriate level of expertise to confirm that it is of an acceptable scientific standard.

# Peer review: Reviewer response for version 1

**DOI:** 10.5256/f1000research.125034.r151763

**Published:** 2022-11-17

**Authors:** Darren Wan-Teck Lim

**Affiliations:** 1 Division of Medical Oncology, National Cancer Centre Singapore, Singapore, Singapore

The authors have completed a manuscript that describes common oncogenes found in lung cancer patients in North Sumatra.

 It is a descriptive and retrospective study.

 Some comments on the data presented have to be made:

1. Only TP53 and EGFR mutations were found in the study. Were the TP53 and EGFR mutations co-mutant in the same patient tumor? And if so how many of them demonstrated co-mutation? Populations with co-mutations of EGFR/p53 do worse than EGFR alone populations and has been described before in other literature. It would be good for the authors here to look into this.
2. Were the Stage IV EGFR mt patients treated with EGFR TKI? If so with which generation of drug and for how long? In other words what is the median OS for the pts who were treated with EGFR TKI vs those who did not receive EGFR TKI? 
3. If 71.25% of the patients were stage IVA, how were the other stages of disease treated? Did stage IIIB vs IIIC vs IV survive differently? and what were the proportion of pts in each stage who were treated with EGFR TKI? 

Is the work clearly and accurately presented and does it cite the current literature?

Yes

If applicable, is the statistical analysis and its interpretation appropriate?

Yes

Are all the source data underlying the results available to ensure full reproducibility?

Yes

Is the study design appropriate and is the work technically sound?

Partly

Are the conclusions drawn adequately supported by the results?

Partly

Are sufficient details of methods and analysis provided to allow replication by others?

Yes

Reviewer Expertise:

Lung cancer

I confirm that I have read this submission and believe that I have an appropriate level of expertise to confirm that it is of an acceptable scientific standard, however I have significant reservations, as outlined above.

# Response

**Published:** 2022-12-22

**Authors:** Johan Samuel Sitanggang

**Affiliations:** Universitas Sumatera Utara, Indonesia

Dear reviewer,

 Thank you for the reviews that have been submitted to us. Meanwhile, we also would like to convey a number of things related to several reviews and questions that have been made by the reviewer as follows.

 1. Were the TP53 and EGFR mutations co-mutant in the same patient tumor? And if so how many of them demonstrated co-mutation?

 In this study, there were no samples with co-mutation P53 and EGFR at the same time. Only co-mutations of exon-19 and exon-21 were found in the EGFR found in 2 individuals. This has also been discussed in the discussion column of this study, precisely in paragraph 2 page 6 of this study, where based on previous studies, a higher number of mutations in EGFR is associated with a worse prognosis. However, in this study, better overall survival and progression free survival were found in patients with double mutations compared to single mutations. This might happen because the number of samples with double-mutation in the study was very small. It is known that the co-mutation of P53 and EGFR is associated with a worse patient prognosis, this has been found based on the available literature, we may add this to the discussion of this paper.

 2. Were the Stage IV EGFR mt patients treated with EGFR TKI? If so with which generation of drug and for how long? In other words what is the median OS for the pts who were treated with EGFR TKI vs those who did not receive EGFR TKI?

 Because of the objectives of this study did not cover this, accompanied by limited data regarding patients and also the lack of research samples so we could not present the information that reviewer asked. Based on previous studies, it was found that there was an increase in the prognostic parameters of EGFR mt patients with treatment with EGFR-TKI, with KRAS mutations found playing a major role in the existence of resistance to this drug. In our next study, we are going to make a research in this regard. Thank you for the advice that has been given.

 3. If 71.25% of the patients were stage IVA, how were the other stages of disease treated? Did stage IIIB vs IIIC vs IV survive differently? and what were the proportion of pts in each stage who were treated with EGFR TKI?

 This study focuses only on assessing the role of oncogenes and tumor suppressor genes from lung cancer on survival rates of lung cancer patients in North Sumatra, Indonesia, so that in this paper we do not include data on the management of each patient. Previous studies have indeed mentioned several specific treatments for different mutations. However, we will probably consider this in the implementation of our next research.

 We would probably publish a second version soon adding some of the suggestions you have provided. Thank you for the review you have given.

# Peer review: Reviewer response for version 1

**DOI:** 10.5256/f1000research.125034.r145779

**Published:** 2022-08-11

**Authors:** Fariz Nurwidya

**Affiliations:** 1 Department of Pulmonology and Respiratory Medicine, Universitas Indonesia-Pesahabatan Hospital, Jakarta, Indonesia

In this study, the author determined the role of KRAS, EGFR, and TP53 mutations in the survival rate of lung cancer patients in the population of North Sumatra, Indonesia. They assessed the expressions of EGFR, RAS, and TP53 protein from lung cancer specimens. Furthermore, they performed sequencing analysis to detect the mutations of EGFR exon 19 and 21, RAS protein exon 2, and TP53 exon 5-6 and 8-9. Finally, survival rate analysis was presented with Kaplan Meier. They found that EGFR mutations showed a higher survival rate, while TP53 mutations showed a lower survival rate in overall survival and progression-free survival.

 This is a well-written manuscript and provides data on oncogenes and tumor suppressor gene mutations in Indonesia and the relationship with the survival rate.

Is the work clearly and accurately presented and does it cite the current literature?

Yes

If applicable, is the statistical analysis and its interpretation appropriate?

Yes

Are all the source data underlying the results available to ensure full reproducibility?

Yes

Is the study design appropriate and is the work technically sound?

Yes

Are the conclusions drawn adequately supported by the results?

Yes

Are sufficient details of methods and analysis provided to allow replication by others?

Yes

Reviewer Expertise:

Thoracic oncology.

I confirm that I have read this submission and believe that I have an appropriate level of expertise to confirm that it is of an acceptable scientific standard.
